# Identifying Epilepsy Based on Deep Learning Using DKI Images

**DOI:** 10.3389/fnhum.2020.590815

**Published:** 2020-11-09

**Authors:** Jianjun Huang, Jiahui Xu, Li Kang, Tijiang Zhang

**Affiliations:** ^1^College of Electrical and Information Engineering, Shenzhen University, Shenzhen, China; ^2^Imaging Department, The Affiliated Hospital of Zunyi Medical University, Zunyi, China

**Keywords:** epilepsy, diffusion kurtosis imaging, deep learning, hippocampus, MRI-negative

## Abstract

Epilepsy is a serious hazard to human health. Minimally invasive surgery is currently an extremely effective treatment to refractory epilepsy. However, it is challenging to localize the lesion for most patients because they are MRI negative. The identification of epileptic foci in local brain region will be helpful to the localization of epileptic foci because we can infer whether there is a lesion from the results of the classification. For the sake of simplicity and the data we collected, only the hippocampus was segmented as a local brain region and classified in this paper. We recruited 59 children with hippocampus epilepsy and 70 age- and sex-matched normal controls, and diffusion kurtosis images (DKI) for all subjects were collected because DKI can understand the pathological changes of local tissues and other regions of epileptic foci at the molecular level. Then, a mask of hippocampus was made to segment the hippocampus of FA, MD, and MK images for all subjects, which are the parameter images of DKI and were used to perform the independent-sample *t*-test and the classification task. At last, a convolutional neural network (CNN) based on transfer learning technique was developed to extract features of FA, MD, MK, and the fusion of FA and MK, and support vector machine was employed to classify epilepsy and normal control. Finally, the classifier produced 90.8% accuracy for patient vs. normal controls. Experimental results showed that the features extraction based on CNN is very effective, and the high accuracy of classification means that FA and MK are two remarkable features to identify epilepsy, which indicates that DKI images can act as an important biomarker for epilepsy from the point of view of clinical diagnosis.

## Introduction

Epilepsy is a chronic disease caused by the sudden abnormal discharge of neurons in the brain, which leads to transient brain dysfunction. As its prevalence continues to rise, epilepsy has gradually become the second common neurological disease after headache.

Epilepsy often occurs in adolescents, and it will greatly affect the patient’s life if not treated in time (Hoare and Russell, 2010). The younger the patient, the greater the impact. Epilepsy may cause lifelong brain dysfunction in children and even endanger life (Harvey et al., 2010). The early diagnosis and localization is extremely important for interventional therapy. Minimally invasive surgery is currently an extremely effective treatment to refractory epilepsy. However, it is challenging to localize the lesion for most patients because they are MRI negative.

Preference of diagnosis for epilepsy is EEG (electroencephalogram), followed by CT and MRI. As the most common means of diagnosis, EEG could be used to determine whether the patient has a seizure and to determine the type of seizure, which can help to identify the inducing factors of epilepsy. However, because EEG lacks the understanding of etiology and low spatial resolution of conventional EEG, the location and microstructural changes of epileptogenic foci cannot be detected. CT and conventional MRI is of great value in the diagnosis of epilepsy, the location of the lesion, the choice of treatment plan, the evaluation of curative effect, and the judgment of prognosis. But unfortunately, both of them do nothing about primary epilepsy without obvious structural changes. It should be noted that about 30% of the epilepsy population are MRI negative (Jörg et al., 2013). Considering the MRI’s technical advantages of being non-invasive, no injury, and fast speed, how to use it to improve the diagnosis accuracy of brain lesions in patients with primary MRI-negative epilepsy is of great significance.

Along with the development of MRI, diffusion-weighted imaging, perfusion-weighted imaging, magnetic resonance spectroscopy (MRS), blood oxygen level dependent, and so on can understand tissue physiology and disease from the point of view of molecular, hemodynamic, and metabolite. They cannot only obtain the information of brain tissue morphology and structure but also describe the functional state of brain from molecular, metabolic micro-angle, and even brain network. At present, these techniques have been widely used in the clinical and scientific research of central nervous system diseases, such as early diagnosis and prognosis evaluation of ischemic cerebral infarction, preoperative grading and postoperative evaluation of brain tumors, and microscopic neuropathological changes of some mental diseases (Zhang, 2012; Liu and Zhang, 2014; Jiang et al., 2020; Kang et al., 2020).

Researchers around the world carried out research aiming to find effective ways to localize epilepsy. Li (2018) reported that the localization rate of MRI-negative epilepsy patients using PET can reach 56.4%, the localization accuracy of EEG combined with fMRI is 61.5 and 63.2%, localization accuracy is achieved using MEG (magnetoencephalography). 3D-arterial spin labeling is also employed by researchers to perform the localization of epilepsy (Sebastiano et al., 2020), and they reported a 69.2% accuracy. At the same time, they found that ^1^H-MRS (proton magnetic resonance spectroscopy) can localize epilepsy with 76.9% accuracy. Furthermore, when combined with the two methods, 84.6% localization accuracy was achieved.

Studies have also shown that diffusion tensor imaging (DTI) and diffusion kurtosis imaging (DKI) have important value in the study of pathophysiological mechanisms of epilepsy, lesion focus localization, and so on (Gao et al., 2015). Gaizo et al. (2017) used machine learning to classify epilepsy based on diffusion MRI, achieving accuracy of 68% (FA), 51% (MD), and 82% (MK), and he also verified statistically that FA and MK are more significant than MD to diagnose epilepsy. Gaizo et al. (2017) did similar work, but what they used to classify was the image of the entire brain region, and they could only judge whether an individual was ill, and could not locate epilepsy lesions. The purpose of this paper is to provide a method to analyze DKI data using deep learning methods to locate conventional MRI-negative epilepsy lesions. The method of segmenting and then classifying the kurtosis of DK images is mainly used to locate epileptic foci in patients with conventional MRI-negative epilepsy. In particular, a convolutional neural network (CNN) is introduced to segment the hippocampus, and VGG16 that has been transfer learned is used to characterize the image. The extraction of vectors uses feature vectors as the input of the support vector machine (SVM) classification network, and the results obtained by our method are much better than those of Gaizo et al. (2017). The accuracy is improved by 19.16% (FA), 8.78% (MD), and 8.81% (MK).

In this paper, DKI images were collected for all subjects, based on which we focused on the classification of epilepsy in a single brain region, specifically, the hippocampus. First, we analyze the sensitivities of FA, MD, MK, and the fusion of FA and MK to the identification of epilepsy by means of statistical method. Then, a CNN with transfer learning technique was developed to extract features for all subjects, which were used to identify epileptic foci from normal control. Finally, 90.8% classification accuracy is achieved, which makes it clinically feasible to identify epilepsy using DKI based on deep learning.

It should be noted that although we have only identified one brain region for epilepsy, actually, the localization of epilepsy foci can be achieved by the results of identifying epilepsy in several other brain regions, which is not discussed here because it is beyond the scope of this paper. In addition, although the hippocampus is not the most common brain region for epileptic foci in children, we only take this as an example to illustrate our method based on the collected data. The analysis of other brain regions can be fully followed by the same analysis method of the hippocampus discussed in this paper, which will contribute to the localization of epileptic foci.

## Materials and Methods

### Subjects

The data in this article are collected by the Affiliated Hospital of Zunyi Medical University from 59 patients (32 males and 27 females) with epilepsy lesions in the hippocampus; all patients were diagnosed according to the 2010 version diagnostic criteria of the ILAE (Kramer, 2010) by intermediate-grade pediatricians or higher. At the same time, 70 healthy volunteers (37 males and 33 females) with matching gender, age, and education level were recruited as the control group. The subjects were all right handed.

The statistics of normal control and epilepsy patients by age are as follows (Table 1).

**TABLE 1:** Corresponding statistical information of subjects.

**Characteristic**	**Patients (*n* = 59)**	**Normal (*n* = 70)**
Gender (M/F)	32/27	37/33
Age (years)	11.13 ± 2.89 (range 7–18)	12.82 ± 3.13 (range 7–18)
Handedness	59R	70R
Duration (years)	4.22 ± 3.15 (range 1–13)	–
VIQ	93.90 ± 18.99 (range 46–122)	–
PIQ	90.87 ± 18.99 (range 43–129)	–
FIQ	91.93 ± 19.36 (range 39–125)	–

*M, male; F, female; R, right; VIQ, verbal intelligence quotient; PIQ, performance intelligence quotient; FIQ, full-scale intelligence quotient.*

### Image Acquisition

All participants underwent MRI examinations (3.0 T HDxt; GE Healthcare, Milwaukee, WI). Before starting the examination, the metal substance carried by the subject was removed, earplugs are worn to reduce noise, the subject’s head was fixed to reduce head movement, and the head was straightened. Participants were asked to close their eyes, lie down, and stay awake. The scanning range is the entire head.

Scanning parameters:

1. 3D T1BRAVO: repetition time = 7.8 ms, echo time = 3.0 ms, inversion time = 450 ms, flip angle = 15°, field of view = 256 × 256 mm, spatial resolution = 1 × 1 × 1 mm, slice thickness = 1 mm, slices = 256, scan time = 208 s.
2. DKI: b value (0, 1,000, 2,000 s/mm^2^), the diffusion-sensitive gradient field is applied in 50 directions, b value is 0, scan 2 times, field of view (FOV): 240 × 240 mm, spatial resolution: 1 × 1 × 4 mm; echo time (TE): 100 ms; repetition time (TR): 10,000 ms; layer thickness: 4 mm, layer spacing: 0 mm; flip angle (FA): 90°, scan time: 8′50′′, scan layer number: 35 layers, a total of 1820 images were collected in the whole brain.

### Data Preprocessing

All data have been preprocessed by a series of standard preprocessing procedures. dcm2niigui software was used to convert all the image formats from Dicom to 3D nifty. Because children’s brain structure is significantly different from that of adults, the common adult brain template cannot be used to perform the registration. Instead, a child’s brain is selected as a template from our data. We used SPM8 toolbox (statistical parametric mapping 8)^1^ in the MATLAB R2017a platform to register all data.

**FIGURE 1:**
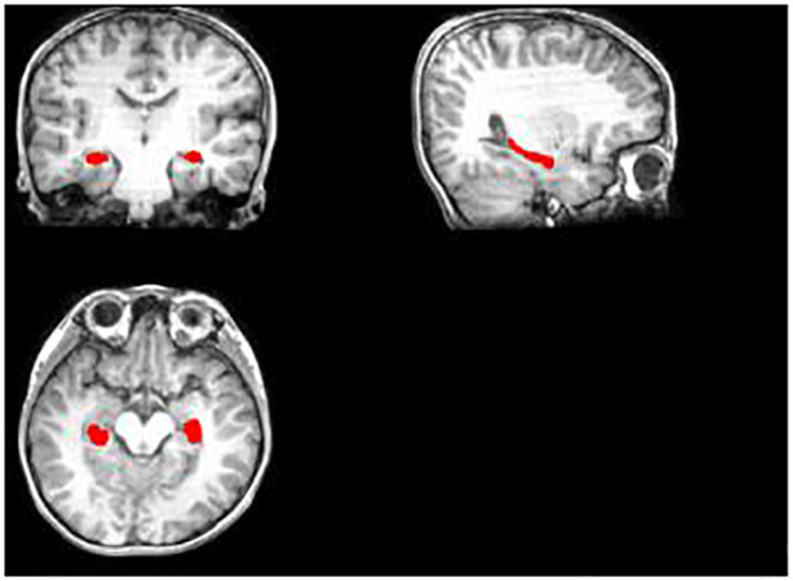
The segmented hippocampus in T1.

**FIGURE 2:**
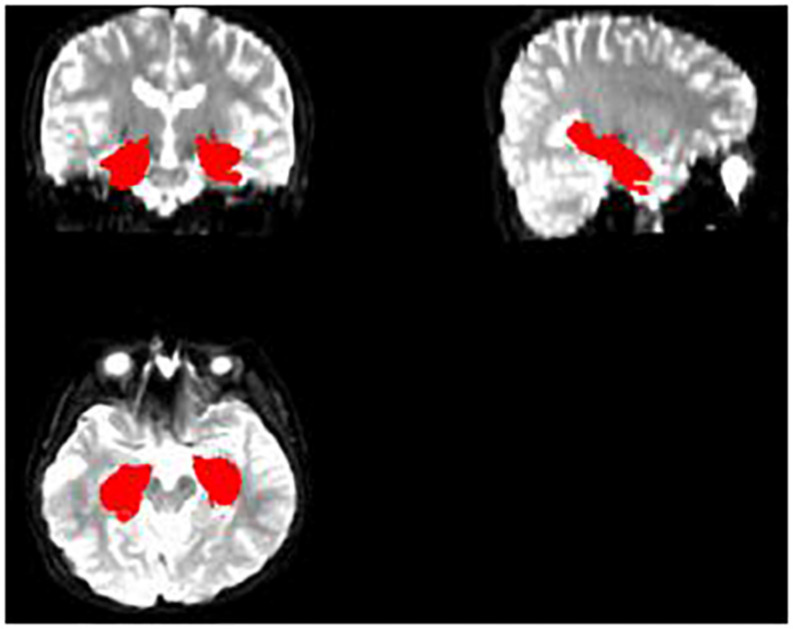
The completed hippocampus mask.

Then, we tried to make a hippocampus mask. First, we segmented the hippocampus region for each subject’s T1 image, which was used to mask the hippocampus region of FA, MD, and MK maps after some preprocessing (Figures 1, 2). Deep segmented CNN was employed to perform the segmentation which was trained by Ataloglou et al. (2019). Then, because it contains T1 and segmented hippocampus images, the EADC-ADNI HarP dataset^2^ was used to fine-tune the network and segment the hippocampus region of T1 image for each subject. To fully cover the hippocampus area for all subjects, union operation was used to produce the hippocampus mask. Because it was found that there was little difference between the three age groups in the shape and size of the hippocampus, the hippocampus regions segmented by all subjects are used to calculate the hippocampus mask regardless of the age group.

**FIGURE 3:**
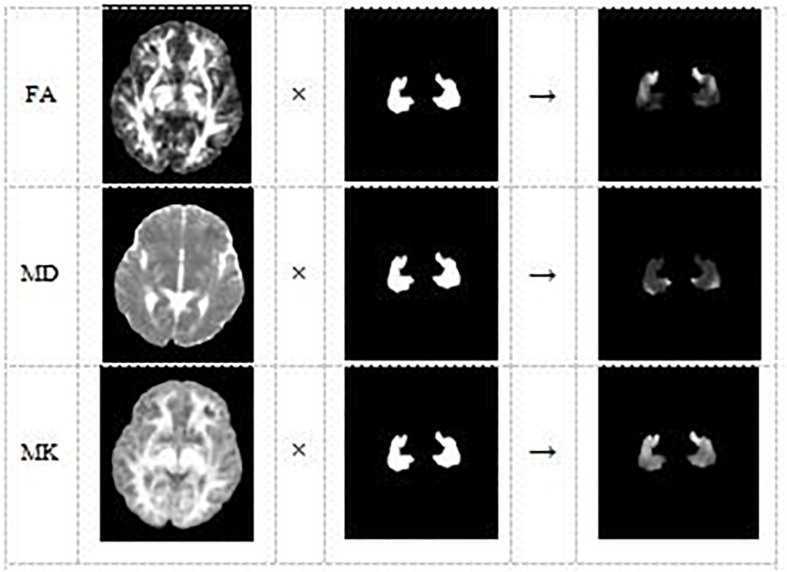
The DKI parameter image of each subject and the hippocampus mask are combined to obtain the hippocampus area of the DKI parameter image of the subject.

Lastly, DKE^3^ software was used to estimate the DKI parameter maps FA, MD, and MK. With the SPM8 toolbox, the affine matrix was calculated from the B0 image of the subject and the T1 image after each registration, and affine transformation and interpolation of the DKI parameter image was performed (Figure 3). Consequently, we can use the hippocampus mask, obtained from the aforementioned operation, to extract the hippocampus regions of all the parameter images FA, MD, and MK. Because the original format of the hippocampus parameter image is 3D nifty, we converted the hippocampus parameter images into jpg format to facilitate feature extraction using deep learning model VGG16.

**FIGURE 4:**
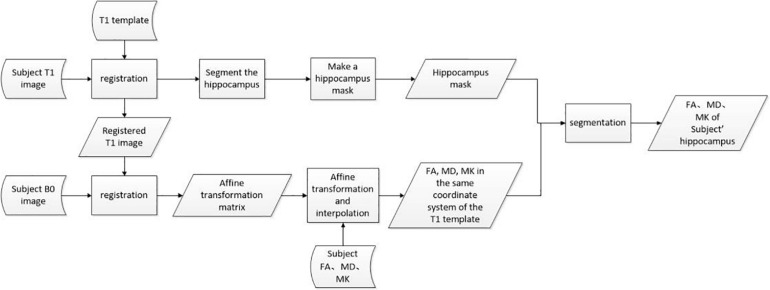
Data preprocessing flow chart.

The aforementioned data preprocessing process can be represented by Figure 4, where rectangles represent operations, parallelograms represent internal data, and arc-shaped quadrilaterals are used to represent external input data.

### Feature Extraction and Classification

#### Feature Extraction With VGG16

Deep learning is a powerful computing method that can automatically learn features and patterns from mass data, and CNN is a kind of deep learning algorithm that has proven to be very effective in classification. In recent years, CNN has been successfully applied to image classification, image recognition, natural language processing, speech recognition, and biological signal processing (Smirnov et al., 2014; Palaz et al., 2015; Yin et al., 2017; Ganapathy and Peddinti, 2018; Shang et al., 2018; Wei et al., 2018; Bird et al., 2020). However, medical image datasets are usually small, which is not suitable for processing with deep neural network directly. To solve this problem, transfer learning technique was introduced in this paper.

First, we transferred pre-trained parameters trained on natural image dataset *Imagenet* with 1000 categories into VGG16 network. Then, the VGG16 network was fine-tuned with transferred pre-trained parameters by the EADC-ADNI HarP dataset because it has the same data structure as the data in this paper. Finally, three VGG16 networks were trained with FA, MD, and MK slice-level dataset, respectively, and the trained VGG16 networks were used to extract features of FA, MD, MK, and the fusion of FA and MK images.

**FIGURE 5:**
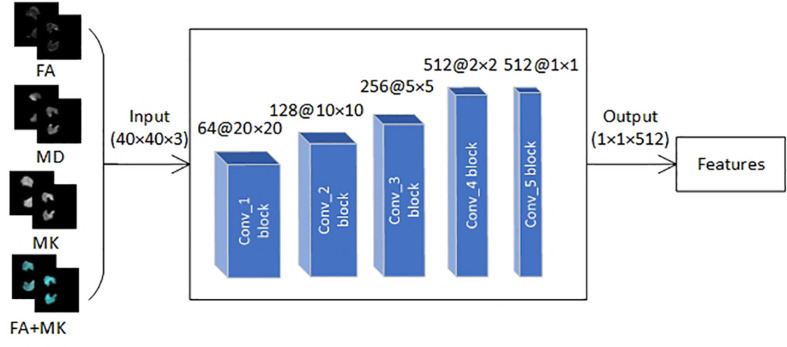
The framework with VGG16 proposed in this paper.

Transfer learning technique is to utilize the pre-trained weights to initialize own network whose structure is the same as pre-trained model trained by a much larger dataset, thus realizes self-adaption from source to target domain. In this study, we transferred VGG16 pre-trained weights trained by nature image dataset Imagenet with 1,000 categories into own VGG16 network (Figure 5). Although brain image is very different from nature images, the first few layers in CNN can extract many generic features, such as side, angle, color, and so on. According to the difference of category number and image attribute between source domain and target domain, we replaced fully connected layer and freezed the pre-trained weights of the first four convolution blocks of VGG16, then the pre-trained weights in the fifth convolution block and the initial weights of fully connected layer were continually updated during training process; the aforementioned courses are also called fine-tuning. The freezed layers will be used to extract generic feature whereas the fine-tuned layer extracts high-level target-specific features.

In feature extraction part, some studies directly use square instead of fine segmented brain region. The reason we do not do that is that the finely divided brain regions cannot only remove interference information but also effectively reduce the amount of calculation and save network running time. Moreover, the purpose of this experimental method is to locate the epileptic foci in patients with conventional MRI-negative epilepsy. If the square brain area is used directly, it will be difficult to determine which specific brain tissue the epileptic foci came from.

### Classification With SVM

We used support vector machine (SVM) to identify epilepsy from normal controls (NC) because of its superior performance in many similar classification tasks. SVM is widely used in classification at present because it can choose the best interface among many classification surfaces and add a penalty factor in the learning process of small samples to produce a certain soft boundary, which can effectively avoid over-fitting. So, it is very suitable for small sample classification task (Bishop, 2006).

In SVM, assume the hypothesis space, namely the linear binary classifier:

(1) $$f\,\left(x\right)=s\,i\,g\,n\,\left(w^{T}\,x+b\right)$$

using marginmin to denote the minimum geometric distance from the hyperplane in the training sample. To maximize the minimum geometric distance, it is equivalent to finding the following function:

(2) $$\arg\,\max_{w}\left(\min_{n}\frac{y\,\left(w^{T}\,x_{n}+b\right)}{w\,}\right)$$

Because the minimization problem has nothing to do with the value of | |*w*| |, we can put | |*w*| | in the aforementioned formula:

(3) $$\arg\,\max_{w}\left(\frac{1}{w\,}\,\min_{n}y\,\left(w^{T}\,x_{n}+b\right)\right)$$

The inner layer minimization problem becomes the minimization function interval, where the function interval after minimization is fixed to 1, then the original problem becomes

(4) $$\begin{array}{c} \arg\,\max_{w}\frac{1}{w\,}\\ s.t.y\,\left(w^{T}\,x_{n}+b\right)\geq 1,n=1,2,\ldots ,N \end{array}$$

According to the usual practice, the maximization problem is transformed into the minimization problem. To facilitate the solution, the second norm is changed to the inner product of the vector, and the basic form of the SVM model is finally obtained, namely:

(5) $$\begin{array}{c} \arg\,\min_{w}\frac{1}{2}\,w^{T}\,w\\ s.t.y_{n}\,\left(w^{T}\,x_{n}+b\right)\geq 1,n=1,2,\ldots ,N \end{array}$$

## Experiments and Results

### Data Preparation

To reduce the interference introduced by irrelevant information and improve the computational efficiency, 36 slices from 40 to 75 were selected in the experiment, each of which has a resolution of 256 × 256. Because the proportion of the hippocampus is too small for data analysis and classification, furthermore, all the slices were cropped to the size of 40 × 40.

All slices were used to build two datasets, which consist of 2,520(70 × 36) epilepsy slices and 2,124(59 × 36) NC slices. The slice-level dataset was split into train set and validation set with the ratio of 8:2. Another subject-level dataset was built for SVM classification, which consists of 129 folders, each of which contains all slices for one subject.

The extracted features were put into the SVM with Gaussian kernel function (rbf) to distinguish the hippocampus from NC.

### Statistical Analysis

In this section, we analyze the two datasets from a statistical perspective. First, we calculated the average gray for all FA, MD, and MK parameter images with non-zero gray values, and IBM SPSS 24 software was used for statistical analysis for MK, FA, and MD of case group and NC group. Table 2 shows the results of independent-sample *t*-test.

**TABLE 2:** Result of independent-sample *t*-test.

	**Normal (× 10^–3^ mm^2^/s)**	**Patient (× 10^–3^ mm^2^/s)**	***p***
FA	0.3288 ± 0.0415	0.2254 ± 0.0208	**0.000**
MD	0.6488 ± 0.0586	0.6538 ± 0.0497	0.603
MK	0.8781 ± 0.0416	0.6978 ± 0.0350	**0.000**

*The bold value represents the result of p < 0.05, indicating that the data has a significant difference.*

It could be found that the mean values of FA and MK of the patients are significantly lower than those of NC, and there is not much difference in the mean value of MD. It is consistent with the DKI parameter image of epilepsy patients proposed in literature (Xia, 2018), where the FA and MK decrease in the location of the lesion. These areas with reduced FA and MK may reflect a lack of myelin sheath, increased axonal membrane permeability, or a less tight neural network (Zhang et al., 2016). It shows that DKI parameters are sensitive to microstructural changes of epilepsy tissues.

It could also be seen from Table 2 that the significance (two-tailed) of FA and MK are both 0.000, and *p* is the significant two-tailed. Because *p* < 0.05, it shows that there is a significant difference between the patient and the NC’s FA and MK. The significance of MD (two-tailed) is greater than 0.05, which indicates that there is no significant difference in MD between patients and NC.

The results are also consistent with that of Wieshmann et al. (1999) and Liacu et al. (2010) using DTI, which can be inferred that the loss of hippocampus neurons is not sufficient to induce significant changes in the arrangement of water molecules in the extracellular matrix. The decrease of FA and the normality of MD indicate the loss of directionality of cell tissue and the integrity of cell density.

**FIGURE 6:**
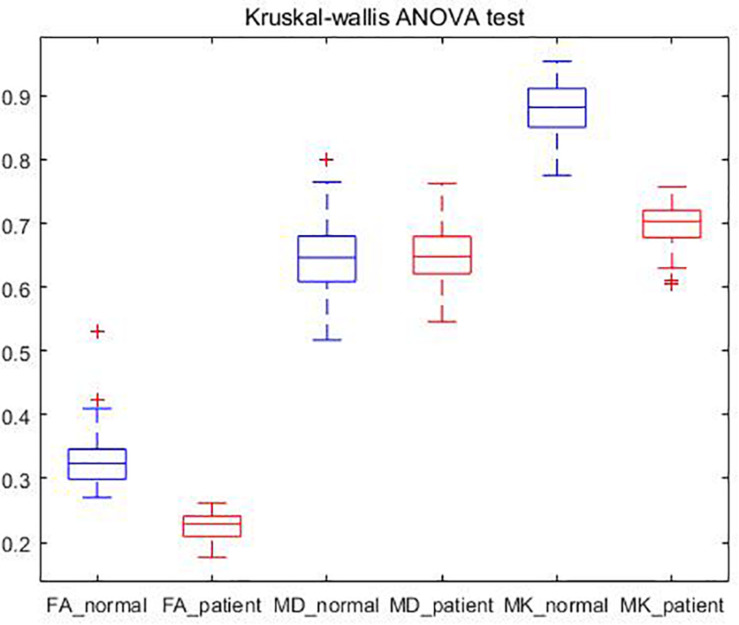
Kruskal–Wallis ANOVA test.

We also conducted Kruskal–Wallis ANOVA test on the subjects, and obtained the FA, MD, and MK box plots of patients and NC as shown in Figure 6.

### Classification With SVM

**FIGURE 7:**
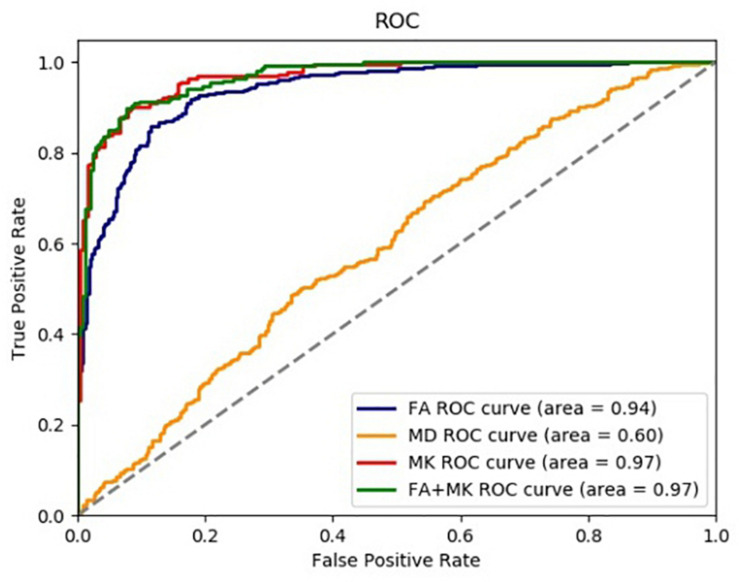
The ROC curve of each test parameter graph in SVM classifier.

We used CNN to extract features and SVM to identify epilepsy from NC in this paper. The accuracy rate (ACC), precision (PRE), sensitivity (SEN), specificity (SPE), the area under the ROC curve (AUC), and the coordinate axis are employed to evaluate the performance of the proposed method. The ROC curves of 5-fold cross-validation are shown in Figure 7. It can be seen from Figure 7 that the AUC value of FA is 0.94, the AUC of MD is 0.60, the MK is 0.97, and the FA + MK is also 0.97. The results indicate that epilepsy patients can be identified from NC effectively by using FA, MK, and the fusion of FA and MK, but not MD. However, there is no significant difference between FA (or MK) and their fusion.

The extracted features are normalized to distributions with mean 0 and variance 1 before feature classification. Then, the normalized features were fed into the optimal SVM classifier selected through exhaustive search. To alleviate the problem of data imbalance, class weights were imposed in SVM classification.

Table 3 lists the results of features classification, including ACC, PRE, SEN, SPE, and AUC in Gaizo et al. (2017) and our approach. As shown in Table 3, the classification performance of our approach is superior to that of Gaizo. An average of accuracy of 90.81%, a sensitivity of 89.29%, a specificity of 93.50%, and an AUC of 91.0% have been achieved for identifying epilepsy from NC with FA, MK, or the fusion of FA and MK. The method using MK achieved the highest accuracy on identifying epilepsy foci in the segmented hippocampus region, and the accuracy using the combination of FA and MK is a little lower than the accuracy 0.9081 achieved by only using the MK, which may be because the simple combination of the two parameters does not make good use of their features.

**TABLE 3:** Classification performance comparison with previous studies based on DKI.

		**Train ACC**	**Test ACC**	**PRE**	**SEN**	**SPE**	**AUC**
FA	Gaizo et al.		0.6830		0.6060	0.7520	
	Ours	0.9398	**0.8716**	0.8760	**0.9041**	0.8281	0.9400
MD	Gaizo et al.		0.5140		0.3450	0.6640	
	Ours	0.7901	**0.5978**	0.6207	0.7676	0.3696	0.6000
MK	Gaizo et al.		0.8200		07650	0.8700	
	Ours	0.9596	**0.9081**	0.9090	0.8929	0.9216	**0.9700**
FA + MK	Ours	0.9864	**0.9075**	0.9230	0.8767	**0.9350**	**0.9700**

*The total bold value represents the best result of the indicator in the table.*

## Discussion

In this paper, we presented a method for identifying epilepsy in a single brain region, especially the hippocampus, from normal control using DKI images based on deep neural network. The main reason for the identification of epilepsy by brain regions is that it can be applied to the localization of epileptic foci. To extract the hippocampus regions of FA, MD, and MK for all subjects, we segmented hippocampus of T1 images for all subjects, which were used to produce a hippocampus mask. The hippocampus of FA, MD, and MK are extracted with the mask, which were used for independent-sample *t*-test and classification. The results of independent-sample *t*-test showed that there was no significant difference in MD and significant change in FA and MK between epileptics and normal controls, which is consistent with the existing research findings.

To identify the epileptics from normal controls, a CNN with transfer learning technique was developed to extract features of FA, MD, MK, and the fusion of FA and MK for all subjects in this paper. Finally, we used SVM to perform the classification task by these features and achieved 90.8% accuracy. This result is superior to the methods of similar techniques that have been published so far. There may be two reasons for the high classification accuracy: first, DKI imaging method; second, feature extraction method based on migration deep learning.

It is well-known that the location of epilepsy is very difficult because most patients are MRI negative. This may be because the tissue changes caused by epileptic foci are too small to be detected by conventional MRI. So, effective MRI imaging is the key to locate epileptic foci. DKI, which measures the diffusion movement of non-Gaussian water molecules in biological tissues, is a sensitive indicator of brain microstructure changes and can detect the abnormalities of brain microstructure before the structural image. DKI has shown its important value in pathophysiological mechanism, localization, and localization of epilepsy. So, it is appropriate to introduce DKI as the imaging technique in this paper. From the experimental results, both the statistical analysis and the classification accuracy after feature extraction have also verified the validity of DKI in measuring microstructural changes of epilepsy patients who are MRI negative.

MK represents the average of the dispersion kurtosis along all directions. It is an average indicator of the degree of diffusion limitation of water molecules in the tissue and indirectly reflects the complexity of the tissue structure. In this study, significant differences in MK values were found, and the average MK value of the normal control group was larger than that of the epilepsy group. Kurtosis K is a sign of environmental heterogeneity, the larger the value, the more restricted the non-Gaussian dispersion of water molecules in the bilateral frontal lobe and bilateral outer capsule tissues of the normal group, and the more complex the tissue microstructure; on the contrary, the decrease of MK value in the epilepsy group indicates that patients with epilepsy may have a loose structure of bilateral frontal lobes and bilateral outer capsules. Possible pathological changes include neuronal degeneration and loss of glial cells, which are often prompted by the pathology of current epilepsy cases. FA is another main parameter of DKI, which infers the shape of white matter fibers and the formation of myelin. Although both FA and MK can describe the dispersion of water molecules in tissues, MK may be more sensitive than FA parameters because it does not depend on the spatial orientation of the tissue structure and takes the average of multiple b values in the same direction of gradient. FA is the anisotropy score, which reflects the degree of anisotropy of the dispersion of water molecules. The smaller the value, the more random the dispersion and the more non-directional. There are also significant differences in FA between epileptics and NC in the experiment, and the parameter values in the normal group are larger than those in the epilepsy group. Because of the restriction by myelin or cell membrane, the white matter nerve fibers in normal people are arranged regularly, and the nerve fiber bundles are tightly arranged, in which the dispersion of water molecules has a higher directionality, so the FA value is higher; when the white matter nerve fiber bundles are damaged, such as the disintegration of neuronal myelin sheath or the proliferation of irregular glial cells, it may lead to changes in the dispersion environment of water molecules. In this case, it may be accompanied by a decrease in the diffusion parameter (axial diffusion coefficient) along the axon of the nerve fiber. It suggests that the changes in the values of these parameters may be related to the bending of axons, which needs further verification.

In addition, feature extraction method also contributes to the high classification accuracy. CNN is an effective feature extractor, which has been successfully applied in many occasions. To extract features more effectively, EADC-ADNI HarP dataset, which has the same type of data as that in the paper, was used to fine-tune VGG16. Artificial intelligence–assisted diagnosis is the direction of medical development, and the paper tried to use the artificial neural network to analyze and classify the data intelligently and good results were obtained. This is of great significance for the clinical application of AI, especially in the application of central nervous system diseases.

It should be noted again that the idea of this paper is just a key part of epileptic foci localization. The whole process for locating epileptic foci needs a number of other operations. First, brain T1-weighted images and corresponding DKI images for subjects are required. Then, each ROI are pre-processed as the hippocampus discussed in this paper and classified by the CNN. According to the classified result, we can identify if there is a lesion. The pre-processing process is shown in Figure 4, and the classification CNN can be built as the method discussed in this paper.

It should be noted again that although the experiment was done by segmenting only the hippocampus in this paper because of the data we collected, actually, we can locate epileptic foci by analyzing other suspected brain regions using the same method. In other words, the method proposed in this paper is actually an indirect approach to perform epilepsy localization. If the lesion is located in another brain area, the same method can be used. For example, when we are not sure where the lesion is, we can input the patient’s DKI parameter map into the segmentation convolutional neural network or software, divide the parameter map into multiple brain regions, and then input the images of each brain region into the feature extraction and classification network to make predictions and judgments; the location of the lesion may provide imaging reference for the study of the pathophysiological mechanism of epilepsy. In this way, we can perform the localization of epileptic foci.

## Data Availability Statement

The raw data supporting the conclusions of this article will be made available by the authors, without undue reservation, to any qualified researcher.

## Ethics Statement

The studies involving human participants were reviewed and approved by the Zunyi Medical University medical ethics committee, Zunyi Medical University. Written informed consent to participate in this study was provided by the participants’ legal guardian/next of kin. Written informed consent was obtained from the individual(s), and minor(s)’ legal guardian/next of kin, for the publication of any potentially identifiable images or data included in this article.

## Author Contributions

JH, JX, and LK: conception and design of study. TZ: acquisition of data. JX: analysis and interpretation of data and drafting the manuscript. LK and JH: revising the manuscript critically for important intellectual content. All authors contributed to the article and approved the submitted version.

## Conflict of Interest

The authors declare that the research was conducted in the absence of any commercial or financial relationships that could be construed as a potential conflict of interest.

## Footnotes

1 http://www.fil.ion.ucl.ac.uk/spm/

2 http://adni.loni.usc.edu/

3 https://www.nitrc.Org/projects/dke/
